# The Challenges of Predicting Suicidal Thoughts and Behaviours in a Sample of Rural Australians with Depression

**DOI:** 10.3390/ijerph15050928

**Published:** 2018-05-07

**Authors:** Tonelle Handley, Jane Rich, Kate Davies, Terry Lewin, Brian Kelly

**Affiliations:** 1 School of Medicine and Public Health, University of Newcastle, Callaghan, NSW 2308, Australia; jane.rich@newcastle.edu.au (J.R.); terry.lewin@newcastle.edu.au (T.L.); brian.kelly@newcastle.edu.au (B.K.); 2 Centre for Rural and Remote Mental Health, University of Newcastle, Callaghan, NSW 2308, Australia; kate.davies@newcastle.edu.au; 3 Centre for Resources Health and Safety, University of Newcastle, Callaghan, NSW 2308, Australia; 4 Hunter New England Mental Health, Newcastle, NSW 2300, Australia

**Keywords:** depression, suicide, suicidal ideation, suicide attempt, rural, Composite International Diagnostic Interview

## Abstract

Suicide is a leading cause of death, particularly in rural and remote areas. Although depression is strongly related to both suicidal ideation and attempt, it lacks specificity as a predictor, and little is known about characteristics that increase suicide risk among people with depression. A telephone version of the World Mental Health Composite International Diagnostic Interview explored lifetime depression, suicidal ideation, suicide attempt, and related factors among a community-dwelling sample of rural and remote Australians, selected for an interview based on a screener for psychological distress (100% of those with high distress, 75% of those with moderate distress, and 16% of those with low distress). Of 1051 participants interviewed, 364 reported lifetime symptoms of depression; of these, 48% reported lifetime suicidal ideation and 16% reported a lifetime suicide attempt. While depression severity was a significant correlate of suicidality for both males and females, suicide attempt was significantly more common among females with a younger age of depression onset, and a higher number of psychiatric comorbidities. No additional factors were significant for males. Among rural and remote residents with lifetime symptoms of depression, the identification of suicide risk may be enhanced by considering individual and contextual factors beyond depression severity. Further research focusing on risk factors for males would be beneficial.

## 1. Introduction

In 2016, suicide was the leading cause of death among all people aged 18–44 and the second leading cause of death for those aged 45–54 in Australia [1]. The rates of males dying from intentional self-harm are three times greater than those for females and those people living in remote and very remote Australia are 1.7 times more likely to die by suicide [2]. Current research suggests that the need to further understand suicide behaviour is critical for the health and future of rural communities in Australia.

About 30% of the Australian population live outside of major cities [3]. Regional and rural Australia is frequently faced with challenges impacting the social, economic, and health-related aspects of its communities. Environmental challenges including extreme weather events, particularly drought, have broad implications on agriculture, family businesses, community prosperity and the mental health and wellbeing of community members [4]. Causative links between these environmental factors and health outcomes can be challenging to measure as these phenomena are naturally occurring and often unpredictable. Nevertheless, there are indicators that highlight the differences between health care in rural Australia compared to metropolitan areas. Health service availability is limited, service access and use for those in rural towns is less than their city counterparts, and then there are also individual characteristics that highlight the need for a different public health approach for those in regional and rural Australia. For example, the quality of stoicism remains strong, as does community stigma around mental health and wellbeing [5]. Those in rural and remote areas also have higher access to lethal means, and lower access to emergency services, meaning that suicide attempts are often more likely to be fatal than in urban areas [6]. Understanding the contextual factors connected to rural people’s health and wellbeing is the current challenge faced by those engaged in research, policy and health service delivery for rural Australia.

Factors associated with suicidal behaviours vary greatly depending on a range of socio-cultural and biological factors. Risk factors have, in particular, been found to vary according to sex differences. The Lundby cohort study found that being male and having a history of depression increased the long-term risk of suicide [7]. Payne, Swami and Stanistreet [8] examined gender differences in suicide behaviours and outcomes. They attribute higher rates of completed suicide amongst males, and higher rates of deliberate self-harm amongst females, to the socially constructed, dichotomous roles performed by men and women. Examples include the expectation that men are strong and less likely to access help, and for women the higher likelihood of accessing medical help and access to non-lethal means.

One of the major risk factors for suicide is previous diagnosis of a mental illness, particularly depression [9,10]. While depression is perhaps the strongest indicator of suicide risk, it lacks specificity; about 15% of Australians will experience major depression across their lifetime, however the majority of these people will not engage in suicidal behaviours or die by suicide. Considering the multitude of research showing an association between depression and suicide, it is logical to target suicide prevention strategies towards those experiencing depression. However, strategies to better identify individuals within this population who may be at increased likelihood of suicidal thoughts and behaviours would be beneficial. As is typically the case, the largely exploratory analyses reported in this paper utilized only a limited set of predictors (e.g., socio-demographic characteristics, depression history and severity, comorbid mental illness, and help-seeking). Other potential predictors of suicidality identified in the literature, but not assessed in the current study, include: hopelessness [11], insomnia [12], previous suicide attempts [13], aggression and impulsivity [14], and losing a family member to suicide [15].

Using a sample of rural community-dwelling adults (selected based on their psychological distress scores, as described below), this paper aims to explore the relationship between depression and suicidal thoughts and behaviours, with a focus on lifetime history, specifically individual and diagnostic characteristics that may improve the prediction of suicidal ideation and suicide attempt. Considering the observed differences between males and females, this paper focused on analyses stratified by gender to identify relevant predictors for each of these groups. Based on previous research, it is anticipated that the pattern of predictors will differ by gender, with females demonstrating higher rates of non-fatal suicidality.

## 2. Materials and Methods

### 2.1. Participants

Data were obtained from the Australian Rural Mental Health Study (ARMHS), a longitudinal population study of mental health in rural and remote communities that has been described in detail previously [16]. Participants were selected randomly from the electoral roll, and completed a postal survey at baseline (2007–2009), as well as a follow-up survey at 1, 3 and 5 years post-baseline (2008–2013). The participants included in the current analysis comprised a stratified subsample who completed the World Mental Health Composite International Diagnostic Interview version 3.0 (WMH-CIDI-3.0) [17], which was conducted at baseline and the 3- and 5-year follow-up. Participants were selected for this interview according to their psychological distress scores. After completing a paper-based version of the K10 psychological distress scale [18] as part of the ARMHS postal survey, interviews were offered at baseline to 100% of those with high distress (a K10 score of 25+), 75% of those with moderate distress (scoring 16–24), and one-sixth of those with low distress (scoring 10–15). It was anticipated that this formula would result in interviews being completed by approximately equal numbers of participants in each of the psychological distress categories. At the five-year follow-up, interviews were offered to those meeting this same criteria, as well as those who completed an interview at baseline (regardless of their current level of distress).

During the WMH-CIDI-3.0, participants answer an initial screener section in which they are asked about lifetime symptoms of depression, including whether they have ever experienced any of the following for several days or longer: feeling sad, empty or depressed, feeling very discouraged about life, and losing interest in things you usually enjoy. The current analysis includes participants who answered positively to at least one of these symptoms.

ARMHS was approved by the Human Research Ethics Committees of the Universities of Newcastle (reference: H-145-1105a) and Sydney (reference: 13,069), and the relevant Area Health Services. All participants provided written informed consent at the time of the postal survey, and re-confirmed their consent orally over the telephone before completing the WMH-CIDI-3.0.

### 2.2. Measures

Participants completed a telephone-administered version of the WHM-CIDI-3.0. The WMH-CIDI-3.0 has been shown to have excellent inter-rater reliability, and good validity and test-retest reliability, and is an acceptable method with which to determine lifetime diagnoses using both face-to-face and telephone delivery [19,20,21]. It has previously been validated in an Australian rural population [22]. The WHM-CIDI-3.0 is a standardised diagnostic interview used to assess the presence of a range of mental disorders according to both Diagnostic and Statistical Manual (DSM-IV) and International Classification of Diseases (ICD-10) criteria. The following lifetime modules were completed: suicidal ideation and attempts; unipolar and bipolar major depression, dysthymia and minor depression (“affective disorder”); post-traumatic stress disorder, generalised anxiety disorder, social phobia, agoraphobia, panic attack and panic disorder (“anxiety disorder”); and alcohol or drug abuse or dependence (“substance use disorder”).

### 2.3. Statistical Analysis

Diagnoses of psychiatric disorder were calculated according to the WMH-CIDI diagnostic algorithms using SAS 9.2 (SAS Institute Inc., Cary, NC, USA). Data were analysed using SPSS (version 24; SPSS, Armonk, NY, USA) and Stata (release 11; StataCorp. LP, College Station, TX, USA).

Using lifetime suicidal ideation and lifetime suicide attempt as the two outcome measures, logistic regressions were performed to explore the relationship between depression severity and suicidality for females and males, as well as exploring a range of related characteristics. The performance of these models was evaluated by Receiver Operating Characteristic (ROC) curves; ROC curves graph the true positive rate against the false positive rate for the different possible values of a prediction model. The overall performance of a ROC curve is evaluated by the Area Under the Curve (AUC). The AUC measures the model’s ability to correctly discriminate between two groups (e.g., those with suicidal ideation and those without), with a higher AUC indicating a better performing model. An AUC of 0.50 represents a chance-level association, while an AUC of 1.00 represents perfect discrimination. Statistical comparisons were made between depression-only and expanded models, to determine whether the inclusion of additional characteristics resulted in more accurate prediction of suicidal ideation and attempt.

## 3. Results

Over the three study waves, 1051 diagnostic interviews were completed. Of these, 364 reported lifetime symptoms of depression, and were included in the present analysis (225 baseline surveys, 139 follow-up surveys). Respondents were predominantly female (68.10%), with an average age of 53.59 years (range 18–91). Approximately half (47.84%) of these also reported lifetime suicidal ideation, while 15.85% reported a lifetime suicide attempt. The sample characteristics are shown in Table 1.

### 3.1. Suicidal Ideation

Overall, 254 participants reported experiencing lifetime suicidal ideation (160 females, 94 males). The average number of depression symptoms was 5.99 ± 1.62 overall, and was significantly higher among those with a history of suicidal ideation (6.37 ± 1.47 vs. 5.64 ± 1.64; F_(1, 346)_ = 19.03, *p* < 0.001). As shown in Table 2, the severity of depression was a significant predictor for both females and males, with each additional depression symptom increasing the odds of suicidal ideation by 44% for females and 28% for males.

**Figure 1:**
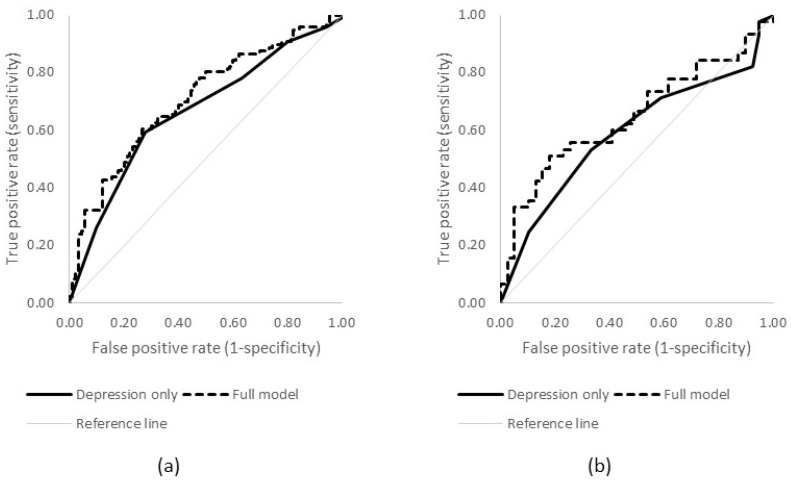
ROC curves predicting lifetime suicidal ideation for (**a**) females and (**b**) males.

However, the ROC analysis showed that, while this effect is statistically significant, its performance as a predictor is limited, producing only moderate AUCs (see Figure 1; females AUC = 0.67, 95% CI 0.58–0.71; males AUC = 0.59, 95% CI 0.47–0.71).

When additional individual and diagnostic characteristics were added into the model, clear differences were observed in the results for males and females. No factors significantly predicted suicidal ideation for males, while among females suicidal ideation was predicted by higher severity of depression, as well as younger age of onset of depression. However, the ROC analyses showed that the predictive value of these models was still limited, resulting in only a small, non-significant improvement over the depression-only models (females AUC = 0.71, 95% CI 0.63–0.78; males AUC = 0.65, 95% CI 0.53–0.77).

### 3.2. Suicide Attempt

**Figure 2:**
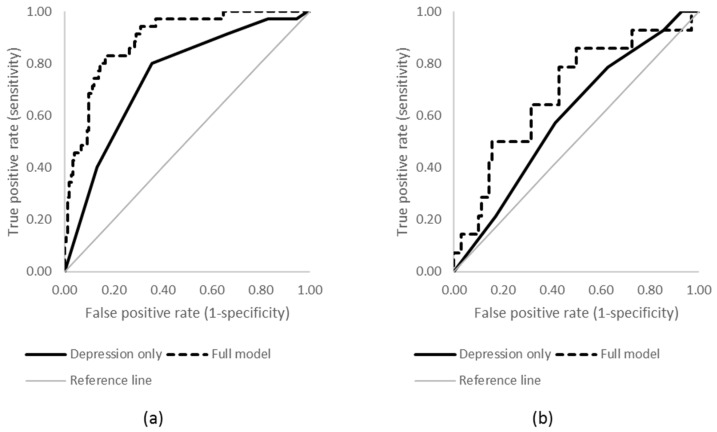
ROC curves predicting lifetime suicide attempt for (**a**) females and (**b**) males.

Overall, 76 participants reported a lifetime suicide attempt (52 females, 24 males). The average number of depression symptoms was significantly higher among those with a history of a suicide attempt (6.84 ± 1.24 vs. 6.14 ± 1.53; F_(1, 165)_ = 8.47, *p* = 0.004). For both males and females, severity of depression was a significant univariate predictor of suicide attempt (see Table 3), with each additional symptom increasing the odds of a suicide attempt by 65% for males and 78% for females. The performance of these models was modest (see Figure 2; females AUC = 0.75, 95% CI 0.66–0.83; males AUC = 0.60, 95% CI 0.44–0.75).

When the additional factors were added into the model, no significant predictors of suicide attempt were observed for males. Similar to the model for suicidal ideation, the odds of a suicide attempt among females were increased among those with higher severity of depression, a younger age of onset of depression, and a higher number of comorbidities. While the expanded model did not improve prediction of the outcome for males (AUC = 0.69, 95% CI 0.53–0.84), for females the model was significantly improved by the inclusion of the additional factors (AUC = 0.89, 95% CI 0.84–0.94; χ^2^_(1)_ = 20.90, *p* < 0.001).

The findings regarding comorbidities were further explored in Table 4. While there was no significant relationship for males between suicide attempt and the number or type of comorbidities, for females several interesting relationships were observed. Over 85% of suicide attempts occurred among females with at least one lifetime comorbidity in addition to depression. In 80% of cases, a lifetime anxiety disorder was reported. Females were significantly more likely to report a lifetime suicide attempt in the presence of a lifetime anxiety disorder, or lifetime anxiety and substance use disorder, compared with a substance use disorder only, or no comorbidities (χ^2^_(3)_ = 66.53, *p* < 0.001).

## 4. Discussion

The findings of this paper have highlighted that, not surprisingly, suicidal behaviours are complex and involve an interplay of clinical, social and individual characteristics. Particularly in rural settings where access to specialised mental health clinicians is limited, understanding contextual and broader aspects of a predictive model may prove vital for suicide prevention. For example, the predictive models for both males and females show that depression is significantly linked to suicidal ideation and attempt, but for females other factors such as age of onset, anxiety, and substance use contribute to a stronger model than depression alone. On the other hand, in males, the complexity continues; other than depression, no factors in the model significantly predicted suicidal ideation or attempt. This lack of significant findings is problematic for the prediction of suicidal thoughts and behaviours among males, and the identification of significant risk factors is vital to reducing suicide rates in this group. Further research with males is needed to better develop this model, however, the results suggest that it is possible that males are more impulsive and spontaneous in their suicidal behaviours than females. Having this understanding of predictive models of suicide for community dwelling rural Australians has the potential to assist in the development of early intervention programs and suicide prevention public health activities. To be able to target the specific populations at risk, and the particular characteristics that expose vulnerability, means that intervention efforts can be tailored to reach such at-risk groups. In rural areas, where there is often a shortage of psychological services, this may mean the development of specific rural-focused resources, such as e-health or other internet-delivered interventions, to assist with the treatment and support of individuals who show increased risk for suicidal behaviours.

### 4.1. The Relationship between Depression and Suicide Risk

This study reinforces research evidence that suggests previous mental illness, particularly depression, is associated with a higher risk of suicide [9,10]. History of suicidal ideation and suicide attempt were both associated with previous experience of depression and this association was stronger when depression was more severe. However, a number of additional factors such as gender and age of onset were also significant. The modelling highlights the complexity of the relationship between suicide risk and depression, the importance of recognising that depression and suicide are not analogous, and the fact that other factors are also important to consider when modelling risk. This is a particularly relevant finding considering that in Australia suicide rates are consistently higher in rural than urban areas, despite there being no notable difference in the prevalence of depression [23]. This suggests that a range of additional factors are likely to contribute to the relationship between depression and suicide risk in this population. Clinical interventions that target additional factors identified in previous research, such as hopelessness, insomnia, and impulsivity, may be beneficial for future research and practice.

### 4.2. Different Risk Factors for Males and Females

In accordance with international evidence, this study illustrates that the risk factors for suicide ideation and suicide attempt vary greatly between males and females [7,8,24,25]. For females, previous experience of depression was a useful predictor of suicide ideation and suicide attempt. A risk model that considers depression, alongside additional factors such as higher severity of depression, a younger age of onset of depression, and a higher number of comorbidities, particularly anxiety, may be useful in recognising suicide risk amongst females. However, for males, while there were associations between depression symptoms and suicide ideation and suicide attempt, when other factors were considered the associations were seen to be weak and this model is less likely to have value in predicting suicide risk.

This suggests that the factors and contexts that shape male suicide are in general different to those for females. This may be related to the fact that male suicidal behaviours tend to be more impulsive, with less opportunity to identify or respond to risk factors before the behaviour is undertaken [6]. Considering that, in Australia, male suicide fatality rates are three times higher than those for females [1], further research to identify early risk factors for males is vital.

### 4.3. Importance of Early Intervention

While this study illustrates the complexities of a predictive modelling approach to suicide risk, it does reveal important relationships between risk factors that can help shape approaches to suicide prevention and treatment. It builds on international evidence that proposes improved risk modelling could play an important role in prevention, treatment and early intervention for people at risk of suicide [26]. This approach also highlights the complexities of suicide and suicide ideation, predicting such behaviour is not straightforward and goes beyond any previous diagnoses of mental illness [22]. Therefore, intervention not only needs to be early but also needs to be multifaceted, targeting gender, age, and social aspects of one’s life. In rural areas where specialist services are limited, this may involve a collaborative approach from both general practitioners, as well as relevant local social and community groups, to ensure positive community engagement as a component of the prevention or treatment of mental illness. Previous Australian research has identified a relationship between strong social support and a reduced likelihood of depression, and intervention studies that target the quality of social support as a depression prevention approach have been recommended [27].

### 4.4. Limitations

This paper has several limitations. Firstly, due to suicidal thoughts and behaviours being low prevalence events, we were only able to look at lifetime reports rather than more recent occurrences. As a result, it is not possible to ascertain whether depression preceded suicidal thoughts and behaviours, or vice versa. In addition, our data were self-reported, and may therefore be affected by inaccuracies including recall bias. Participants may also have felt uncomfortable discussing suicidal thoughts and behaviours, and chosen not to disclose them. Suicide attempts among males are much more likely to result in fatality than among females, so it is possible that the survivor cohort in the present paper does not represent a “typical” male who engages in suicidal behaviours, and our failure to identify significant predictors for suicidality among males may be reflective of this. Although our analyses were stratified by gender, we did not have sufficient power to explore interaction effects.

## 5. Conclusions

While depression is highly correlated with suicidal thoughts and behaviours, our findings suggest that among those with lifetime depressive symptoms, the severity of depression has limited predictive value, and exploring other individual and contextual factors may improve the identification of those at risk of suicidal acts. Further research focusing specifically on males is necessary to identify early warning signs for suicidal behaviours among this group. These may include factors such as hopelessness, insomnia, aggression, and a history of repeated suicidal behaviours.

## Figures and Tables

**Table 1:** Sample characteristics.

Characteristic	Females (*n* = 247)	Males (*n* = 117)
	Mean (SD)	
Age	53.03 (13.01)	55.13 (11.36)
Age of depression onset	28.66 (14.68)	28.18 (14.27)
Number of depression symptoms	6.10 (1.53)	5.85 (1.69)
	% (*n*)	
Help-seeking for depression	87.1 (195)	81.7 (85)
Lifetime anxiety disorder	68.0 (168)	56.4 (66)
Lifetime substance use disorder	27.1 (67)	48.7 (57)

**Table 2:** Regression for suicidal ideation.

Model	Predictor	Females (*n* = 247)		Males (*n* = 117)	
		OR	95% CI	OR	95% CI
Model 1	Depression severity	1.44	1.19–1.74 **	1.28	1.01–1.61 *
Model 2	Depression severity	1.42	1.11–1.80 **	1.13	0.82–1.56
	Age	1.00	0.98–1.03	0.99	0.95–1.03
	Age of depression onset	0.97	0.95–0.99 *	1.00	0.97–1.04
	Comorbidities	1.30	0.82–2.08	1.73	0.90–3.23
	Previous help-seeking				
	No	1.00	-	1.00	-
	Yes	1.10	0.42–2.93	1.68	0.50–5.60

Note: * *p* < 0.05, ** *p* < 0.01. Model also controls for study wave.

**Table 3:** Regression for suicide attempt.

Model	Predictor	Females (*n* = 247)		Males (*n* = 117)	
		OR	95% CI	OR	95% CI
Model 1	Depression severity	1.78	1.29–2.45 **	1.65	1.07–2.54 *
Model 2	Depression severity	1.94	1.25–3.00 **	1.36	0.83–2.23
	Age	0.99	0.96–1.03	0.97	0.91–1.02
	Age of depression onset	0.92	0.88–0.97 **	1.00	0.95–1.05
	Comorbidities	3.78	1.74–8.18 **	0.64	0.27–1.53
	Previous help-seeking				
	No	1.00	-	1.00	-
	Yes	0.80	0.08–8.25	1.58	0.36–7.00

Note: * *p* < 0.05, ** *p* < 0.01. Model also controls for study wave.

**Table 4:** Proportion of suicide attempts by comorbidity.

Comorbidity	Females	Males
No comorbidity	13.5%	29.2%
Anxiety disorder only	30.8%	20.8%
Substance use disorder only	5.8%	16.7%
Anxiety and substance use disorder	50.0%	33.3%

## References

[1] Australia Bureau of Statistics (ABS) Suicide in Australia. Causes of Death, Australia 2017. http://www.abs.gov.au/ausstats/abs@.nsf/Lookup/bySubject/3303.0~2016~MainFeatures~Intentionalself-harm:keycharacteristics~7.

[2] Australian Institute of Health and Welfare (AIHW) (2017). Rural and Remote Health.

[3] Australian Bureau of Statistics (2012). Geographic Distribution of the Population. http://www.abs.gov.au/ausstats/abs@.nsf/Lookup/bySubject/1301.0~2012~MainFeatures~Geographicdistributionofthepopulation~49.

[4] Horton G., Hanna L., Kelly B. (2010). Drought, drying and climate change: Emerging health issues for ageing Australians in rural areas. Australas. J. Ageing.

[5] Judd F., Jackson H., Komiti A., Murray G., Fraser C., Grieve A., Gomez R. (2006). Help-seeking by rural residents for mental health problems: The importance of agrarian values. Aust. N. Z. J. Psychiatry.

[6] National Rural Health Alliance (NRHA) (2009). Suicide in Rural Australia.

[7] Brådvik L., Mattisson C., Bogren M., Nettelbladt P. (2008). Long-term suicide risk of depression in the Lundby cohort 1947–1997—Severity and gender. Acta Psychiatr. Scand..

[8] Payne S., Swami V., Stanistreet D.L. (2008). The social construction of gender and its influence on suicide: A review of the literature. J. Men's Health.

[9] Rihmer Z. (2007). Suicide risk in mood disorders. Curr. Opin. Psychiatry.

[10] Värnik P. (2012). Suicide in the world. Int. J. Environ. Res. Public Health.

[11] Ribeiro J.D., Huang X., Fox K.R., Franklin J.C. (2018). Depression and hopelessness as risk factors for suicide ideation, attempts and death: Meta-analysis of longitudinal studies. Br. J. Psychiatry.

[12] Wojnar M., Ilgen M.A., Wojnar J., McCammon R.J., Valenstein M., Brower K.J. (2009). Sleep problems and suicidality in the National Comorbidity Survey Replication. J. Psychiatr. Res..

[13] Haukka J., Suominen K., Partonen T., Lönnqvist J. (2008). Determinants and outcomes of serious attempted suicide: A nationwide study in Finland, 1996–2003. Am. J. Epidemiol..

[14] Gvion Y., Apter A. (2011). Aggression, impulsivity, and suicide behavior: A review of the literature. Arch. Suicide Res..

[15] Runeson B., Åsberg M. (2003). Family history of suicide among suicide victims. Am. J. Psychiatry.

[16] Kelly B.J., Stain H.J., Coleman C., Perkins D., Fragar L., Fuller J., Lewin T.J., Lyle D., Carr V.J., Wilson J.M. (2010). Mental health and well-being within rural communities: The Australian Rural Mental Health Study. Aust. J. Rural Health.

[17] Kessler R.C., Ustun T.B. (2004). The World Mental Health (WMH) survey initiative version of the World Health Organization (WHO) Composite International Diagnostic Interview (CIDI). Int. J. Methods Psychiatr. Res..

[18] Kessler R.C., Andrews G., Colpe L.J., Hiripi E., Mroczek D.K., Normand S.L., Walters E.E., Zaslavsky A.M. (2002). Short screening scales to monitor population prevalences and trends in non-specific psychological distress. Psychol. Med..

[19] Allen K., Cull A., Sharpe M. (2003). Diagnosing major depression in medical outpatients. Acceptability of telephone interviews. J. Psychosom. Res..

[20] Rohde P., Lewinsohn P.M., Seelet J.R. (1997). Comparability of telephone and face-to-face interviews in assessing Axis I and II disorders. Am. J. Psychiatry.

[21] Haro J.M., Arbabzadeh-Bouchez S., Brugha T.A., De Girolamo G., Guyer M.E., Jin R., Lepine J.P., Mazzi F., Reneses B., Vilagut G. (2006). Concordance of the Composite International Diagnostic Interview Version 3.0 (CIDI 3.0) with standardized clinical assessments in the WHO World Mental Health Surveys. Int. J. Methods Psychiatr. Res..

[22] Handley T.E., Inder K.J., Kay-Lambkin F.J., Stain H.J., Fitzgerald M.N., Lewin T.J., Attia J.R., Kelly B.J. (2012). Contributors to suicidality in rural communities: Beyond the effects of depression. BMC Psychiatry.

[23] Caldwell T.M., Jorm A.F., Dear K.B.G. (2004). Suicide and mental health in rural, remote and metropolitan areas in Australia. Med. J. Aust..

[24] Nock M.K., Borges G., Bromet E.J., Cha C.B., Kessler R.C., Lee S. (2008). Suicide and suicidal behavior. Epidemiol. Rev..

[25] Oquendo M.A., Bongiovi-Garcia M.E., Galfalvy H., Goldberg P.H., Grunebaum M.F., Burke A.K., Mann J.J. (2007). Sex differences in clinical predictors of suicidal acts after major depression: A prospective study. Am. J. Psychiatry.

[26] Yoshimasu K., Kiyohara C., Miyashita K., The Stress Research Group of the Japanese Society for Hygiene (2008). Suicidal risk factors and completed suicide: Meta-analyses based on psychological autopsy studies. Environ. Health Prev. Med..

[27] Werner-Seidler A., Afzali M.H., Chapman C., Sunderland M., Slade T. (2017). The relationship between social support networks and depression in the 2007 National Survey of Mental Health and Well-being. Soc Psychiatry Psychiatr. Epidemiol..

